# Enabling and embedding circularity goals in housing cooperatives

**DOI:** 10.1016/j.rcradv.2025.200272

**Published:** 2025-09

**Authors:** Wim Van Opstal, Nancy Bocken, Jan Brusselaers

**Affiliations:** a Unit of Sustainable Materials and Chemistry, VITO, Boeretang 200, 2400 Mol, Belgium; b Maastricht Sustainability Institute, School of Business and Economics, Maastricht University, Tapijn 11 Building D, P.O. Box 616, 6200 MD Maastricht, the Netherlands; c Institute for Environmental Studies, VU Amsterdam, De Boelelaan 1111, 1091 HV Amsterdam, the Netherlands

**Keywords:** Governance, Circular economy, Circular society, Circular business models, Market failures, Just transition

## Abstract

Shifting towards a circular economy in the built environment is considered an important step toward fostering environmentally sustainable and socially resilient cities. Housing cooperatives, established to provide affordable and democratically governed housing, may offer structural advantages for embedding circularity - but their role in circular transitions remains underexplored. This study investigates how cooperative governance may influence the implementation of circular strategies, including circular design, product-service systems, and shared resource models, across different housing types. Drawing on semi-structured interviews with housing professionals, cooperative representatives, service providers, and policymakers, we assess the comparative institutional advantages and limitations of cooperatives in enabling circular transitions. Our findings indicate that housing cooperatives can mitigate market failures and overcome split incentives through collective ownership, long-term planning, and participatory governance. These features help facilitate lifecycle-based investments, bundled procurement, and shared infrastructure. However, cooperatives also face key challenges, including complex decision-making, limited access to finance, and regulatory barriers. This paper contributes to the understanding of alternative housing models for urban sustainability by offering insights into how cooperative-led initiatives can support circular innovation. It identifies boundary conditions for aligning stakeholder perspectives and embedding circular strategies within cooperative housing, helping to inform inclusive, community-based responses to climate and resource challenges.

## 1 Introduction

Housing plays a critical role in the transition to a circular economy, given its significant environmental footprint. The construction and operation of buildings account for over 40 % of total waste by volume and represent the largest share of global resource consumption (Circle Economy, 2024). Recognising this impact, the Circular Economy Action Plan of the European Commission has identified the building sector as a priority area for circular interventions (European Commission, 2020). As one of the four key urban systems, alongside mobility, products, and food, housing is pivotal in addressing resource efficiency and emissions reduction (Marchesi and Tweed, 2021). However, transitioning to circular housing requires a broader perspective that extends beyond material and technological innovations to include social and governance dimensions. This is especially relevant as affordable housing has been globally recognised as a critical hotspot in sustainable housing, as acknowledged in Sustainable Development Goal 11.1 (United Nations, 2020). Sustainable consumption and production approaches for cities therefore highlight the necessity of engaging non-traditional stakeholders, such as citizen groups, social initiatives, and informal sector representatives, in urban planning and decision-making (Schröder et al., 2019). Social aspects of housing circularity, however, remain relatively underexplored (Vanhuyse et al., 2021).

Housing cooperatives have been established as a model to foster investments in affordable housing, with specific attention to democratic governance and strong stakeholder participation (Türeli, 2022). They are defined as autonomous organisations, collectively owned and democratically governed by their members (ICA, 2012). This means that residents voluntarily unite and purchase shares in the cooperative, granting them specific rights to a dwelling within the cooperative without acquiring ownership of an individual property. Housing cooperatives exist across all continents, though their prevalence and structures vary significantly within and across regions (Lang and Stoeger, 2018). Many operate under rental-based models, where cooperative members receive a discount depending on the number of shares they hold, while other models allow residents to become homeowners (Khatibi, 2023). This housing form is sometimes referred to as a ‘third’ form of housing, offering an alternative to conventional homeownership and rental models, including social housing (Ahedo et al., 2023), or as an alternative to both the capitalist market economy and the state (Brysch et al., 2024).

Housing cooperatives can contribute to a just transition by investing in renewable energy (Lukkarinen et al., 2022) and by translating principles of participatory and distributional justice into possibilities of affordable housing for a broad range of income groups, cultures, and age groups (Aernouts and Ryckewaert, 2019). The way housing cooperatives may enable or embed circular strategies, however, remains a research gap. In this paper, we explore the comparative organisational advantages and disadvantages of housing cooperatives to enable and embed circular strategies. These circular strategies include design-related strategies, circular business models that address total cost of ownership considerations, such as as-a-service models, and sharing models implemented among cooperative members. We consider different housing types and account for stakeholder engagement and alignment, which can be considered key in designing ethical CE interventions in the built environment (Rios et al., 2022). Therefore, we conducted semi-structured interviews with 23 professionals involved in the development, operationalisation, or evaluation of housing cooperatives or housing-related circular strategies. This enables us to investigate how housing cooperatives can enable and embed circularity goals, structured around the following (sub)research questions:

- RQ1: How can cooperative governance influence the implementation of circular strategies in housing?
- RQ2: How does this cooperative difference relate to different circular strategies in different housing types?
- RQ3: How can housing cooperatives align stakeholder positions to implement circular strategies?
- RQ4: What are comparative institutional (dis)advantages of housing cooperatives compared to traditional alternatives in implementing circular strategies?

Belgium provides a relevant setting for this study, as cooperative housing remains a niche model rather than the default housing option, allowing for an external perspective on its potential and limitations in enabling circular housing solutions. Meanwhile, Belgium can be considered as a frontrunner in adopting circular strategies (Claudio-Quiroga and Poza, 2024; D’Adamo et al., 2024). By addressing the institutional conditions shaping cooperative circular housing models, this research contributes to broader debates on governance, economic viability, and regulatory frameworks in the circular built environment.

The rest of this paper is structured as follows. In Section 2, we provide background information on housing and the circular economy, on the role of cooperatives and the circular transition, and on the research gaps we address. In Section 3, we discuss our methods and materials, including the conceptual and empirical framework of this study. In Section 4 we present our results, and in Section 5 we discuss the empirical contributions, policy implications, and limitations of this work.

## 2 Literature

### 2.1 Housing and the circular economy

The transition to a circular housing economy requires systemic change across design, business models, and social practices. A circular transition in housing involves a shift from resource-intensive construction and individual ownership models to approaches that prioritise material efficiency, longevity, and shared use. Design principles are central to enabling circularity in housing. Standardisation of building components facilitates reuse, while design for disassembly and modularity ensures that materials and structures can be repurposed rather than discarded (Arora et al., 2019; Kedir et al., 2023). Procurement policies and building codes that promote recyclability and remanufacturing can further embed circularity in the built environment (Adabre et al., 2022). Additionally, digital tools such as material passports, housing passports, and building information modelling support circular design by improving material traceability and enabling informed decision-making across the construction and renovation phases (Çetin et al., 2022, 2023; Keena et al., 2025). Beyond material considerations, design-related circularity in housing also involves the adaptive reuse of buildings to respond to evolving societal needs. Repurposing large homes into multiple smaller units can extend the functional lifespan of buildings, reducing the need for new construction while answering changing urban needs (Çimen, 2021). Proper design allowing for retrofitting, materials reuse, and recycling can save money and reduce environmental impacts (de Feijter, 2023; Galle et al., 2021), as well as increase the value of properties (Suzuki and Shibata, 2022).

A complementary approach to promote circularity in buildings is shifting from ownership-based housing models to Product-Service Systems (PSS) (Tukker, 2015). In a PSS model, housing-related assets - such as kitchens, façades, or heating systems - are provided as services rather than products, potentially extending product lifetimes and reducing material consumption (Ghafoor et al., 2024). PSS can promote efficiency, longevity and sufficiency in energy, material and space usage levels (Ghafoor et al., 2023). By aligning financial incentives with long-term performance, PSS encourages maintenance, refurbishment, and reuse rather than premature replacement. Performance-based contracts, such as on solar photovoltaics, allow providers to retain ownership of materials, ensuring their optimal use and recovery (Mont, 2002; Van Opstal and Smeets, 2023). However, poorly implemented PSS models risk rebound effects, where increased service availability leads to higher overall resource use (Ackermann and Tunn, 2024; Kjaer et al., 2019). Careful design of service contracts and regulatory oversight is therefore necessary to maximise the circular potential of PSS in housing.

A third approach, sharing, touches upon the crossroads between design and social practices. It may involve shared living spaces, as in co-housing and mixed-use buildings, reducing the need for redundant construction and minimising material demand (Christis et al., 2019). Furthermore, it may include short-term service models, such as sharing tools and equipment, or cars and bicycles (Encarnación et al., 2024; Marchesi and Tweed, 2021). Sharing models offer circular benefits by reducing material and energy consumption per capita (Cherry and Pidgeon, 2018; Cuomo et al., 2021). Digital platforms and smart technologies can further support shared housing models by enabling flexible access to communal spaces and resources (Tunn et al., 2020). However, the success of such initiatives depends on social acceptance and governance structures that enable mutual trust, ensure equitable access, and replicate dynamics that have been taken-for-granted by many generations in informal economies (Holmes, 2018).

### 2.2 Cooperatives and the circular transition

Cooperatives have long played an important role in fostering economic and social resilience, particularly in addressing common-pool resource challenges and market failures (Van Opstal et al., 2025b). Historically, cooperative structures have evolved from guilds, mutual aid societies, and indigenous forms of resource governance, demonstrating the fundamental role of collective organisation in achieving shared goals (Mayer, 2018; Ward, 1995). In modern economies, cooperatives are well-established across diverse sectors, including agriculture, finance, energy, and housing, providing alternatives to investor-owned firms (Hansmann, 1999; Novkovic, 2022). The International Cooperative Alliance (ICA) has formalised a set of cooperative principles that underpin their governance and operations, ensuring that cooperatives remain accountable to their members rather than external shareholders (ICA, 1995). These principles align with broader objectives of sustainability and inclusivity, making cooperatives an organisational form worthwhile to investigate in search of a circular transition (Van Opstal et al., 2025a; Ziegler et al., 2023). As a circular transition requires systemic change, involving collaboration among multiple stakeholders, including consumers, businesses, policymakers, and civil society actors (Schröder et al., 2019), the cooperative model may offer institutional advantages that align with circular objectives. Cooperatives prioritise collective ownership, resource pooling, and democratic governance, enabling the prioritisation of long-term sustainability over short-term profit maximisation (Novkovic, 2008). Moreover, their embeddedness in local communities allows them to foster circularity in regional economies, strengthening socio-economic resilience (Bretos and Marcuello, 2017).

Empirical studies highlight advantages of cooperatives in enabling circularity. For example, recycling cooperatives have been instrumental in integrating informal waste pickers into formal waste management systems, enhancing both social equity and material recovery efficiency (Buch et al., 2021; Valencia et al., 2023). Agro-industrial cooperatives have also demonstrated potential in closing resource loops through symbiotic industrial processes and waste valorisation strategies (Barros et al., 2023). The commitment of cooperatives to member benefits and joint-user value instead of profit-maximisation helps to focus on long-term community goals and reinvesting profits into sustainable initiatives, distinguishing them from conventional firms that often prioritise shareholder returns (Baranchenko and Oglethorpe, 2012; Lafont et al., 2023).

However, cooperatives also face structural constraints. On the one hand, some challenges arise from inherent characteristics of cooperatives, including complexities following from democratic decision-making processes and specific challenges such as the ‘horizon problem,’ which occurs when a member’s residual claim on an asset is shorter than the life of the asset; ‘free-rider problems’ stemming from joint asset ownership; and the risk of ‘demutualisation,’ where growth may lead to a dilution of cooperative identity and member commitment (Novkovic, 2008). On the other hand, cooperatives also face external constraints linked to market structures, financial systems, and regulatory frameworks that are designed around investor-owned enterprises. As cooperatives do not always align with these dominant institutional logics, they may encounter barriers to accessing capital, policy support, and market integration, which can limit their scalability and long-term viability (Royer, 2023).

Recent academic work on circular societies suggests that circular transitions must go beyond technological solutions and embrace governance models that prioritise participatory decision-making and economic decentralisation (Bauwens et al., 2020; Jaeger-Erben et al., 2021). In this context, cooperatives offer a viable institutional framework for embedding circularity in local economies by enabling shared ownership models, reducing market-driven pressures, and fostering democratic participation (Limnios et al., 2018; Puusa et al., 2013). Their ability to internalise externalities, reduce transaction costs, generate economies of scale, and enhance social cohesion render cooperatives a promising organisational model for advancing a circular economy and society (Huybrechts and Mertens, 2014; Ziegler et al., 2023).

### 2.3 Research gap

Despite the growing attention to the CE in the built environment, limited research has explored how cooperative governance structures facilitate or hinder the implementation of circular strategies in housing. This is particularly relevant given the importance of user needs and governance in sustainable housing (Adefila et al., 2020). While previous studies have recognised the role of social housing communities in CE transitions (Marchesi and Tweed, 2021), bottom-up cooperative initiatives remain understudied.

Therefore, we address four research gaps. *Firstly*, by investigating how cooperative governance fosters or challenges circular strategies in housing, this research complements existing literature that predominantly focuses on investor-driven CE initiatives (Geissdoerfer et al., 2023; Hina et al., 2022; Lüdeke-Freund et al., 2019).

*Secondly*, the relationship between cooperative governance and different circular strategies in various housing types also remains unexplored. Housing typologies play a significant role in determining the environmental and material implications of circular construction and renovation (Soonsawad et al., 2024). However, the cooperative model introduces additional complexities, as different governance structures may influence the feasibility and effectiveness of certain CE interventions.

A *third* research gap concerns whether and how housing cooperatives align stakeholder positions to implement circular strategies effectively. Circular business models in housing require the engagement of multiple stakeholders, including residents, local authorities, financial institutions, and service providers (Ghafoor et al., 2024). However, stakeholders often have divergent priorities, with cooperatives seeking to balance economic sustainability with social and environmental goals, while external actors may prioritise financial returns or regulatory compliance (Schröder et al., 2019).

*Finally*, little is known about the comparative institutional (dis)advantages and disadvantages of cooperatives in implementing circular housing strategies, compared to commercial real-estate companies, social housing associations, and public authorities. This comparative perspective is crucial, as cooperative housing models often struggle against conventional homeownership due to entrenched financial and policy frameworks favouring individual ownership (Galle et al., 2021).

## 3 Methods and materials

### 3.1 Conceptual framework

Housing cooperatives typically focus on ensuring long-term affordability by sharing ownership and management responsibilities while prioritising user-value over profit-driven motives. They are fundamentally defined by shared ownership and governance of physical infrastructure, a legal framework, and collective economic responsibility (Avilla-Royo et al., 2021). Moreover, the model of housing cooperatives is distinguished by its adherence to cooperative principles as a governance framework and is often designed to provide affordable, sustainable, and community-oriented housing solutions (Cooperative Housing International, 2024).

Cooperatives, rely on a set of organisational governance principles, defined by the International Cooperative Alliance (ICA) (see Table 1) that define the cooperative difference compared to other organisational forms (ICA, 1995). Cooperative members wear multiple hats, being both owners and users of the firm, which reduces the costs of market contracting under market failures while keeping costs of ownership and collective decision-making low (Hansmann, 1999; Spear, 2000). The set of ICA principles enables them to limit opportunistic behaviour of their members by having strong mechanisms for monitoring and control (Herbst and Prüfer, 2016; Huybrechts and Mertens, 2014), including sociopsychological mechanisms such as reputation, reciprocity, and punishment strategies (Bowles and Gintis, 1998; Ostrom, 2000). These ICA-principles serve as a guiding framework to govern and safeguard the cooperative mission, member participation, and stakeholder interaction (Grimley and Chan, 2023; Novkovic, 2008).

**Table 1:** The cooperative principles (ICA, 1995) and their rationale, applied to housing.

ICA Principle	Rationale, applied to housing
1. Voluntary and open membership	The design of admission and exit criteria is crucial to attracting a balanced mix of residents and ensuring long-term incentives for contributing to the cooperative.
2. Democratic member control	Safeguarding the mission and vision of the housing cooperative to ensure long-term affordability and alignment with the needs of residents.
3. Member economic participation	When members have a financial stake, this may resolve ‘split incentives’ among residents and foster trust and collaboration to contribute technical and experiential knowledge.
4. Autonomy and independence	Housing cooperatives can collaborate with other stakeholders (governments, civil society organisations, etc.) while securing their mission and vision in their statutes.
5. Education, training, and information	Enhances the ability of members to contribute effectively to their cooperative while empowering members to engage in cooperative behaviour and the enforcement of norms of reciprocity.
6. Cooperation among cooperatives	Enhances network reciprocity and a cooperative culture. By collaborating, local cooperatives can retain democratic member control while achieving economies of scale, building countervailing power or fostering market formation when markets are incomplete or missing.
7. Concern for community	Cooperatives are rooted in the communities where they operate, providing a comparative institutional advantage to internalise externalities and address community concerns.

Based on Henrich et al. (2001), Limnios et al. (2018), Novkovic (2008), and Van Opstal et al. (2025a).

Although millions of families worldwide live in housing cooperatives, the model remains relatively obscure in many countries (Cooperative Housing International, 2024). Therefore, we focus on intrinsic design parameters of housing cooperatives and remain agnostic to specific technologies and market parameters (including wages, energy prices, and prices of materials and components). Analytically, this involves insights from law and economics, investigating the comparative institutional advantages of housing cooperatives to achieve economies of scale and resolve market failures.

Regarding economies of scale, we consider supply-side economies of scale, both internal (where higher volumes lead to lower average costs) and external (where a sufficiently developed ecosystem of firms fosters good market and institutional conditions). We also consider demand-side economies of scale, which lead to network effects following from a sufficient number of households to participate in a market (Varian, 2018). Furthermore, we investigate the importance of dynamic economies of scale, which reflects learning effects. Regarding market failures, we investigate the role of externalities, which may result in split incentives between and among residents, owners, and surrounding communities (Bird and Hernández, 2012) of the suboptimal provision of innovation and public goods (Jaffe et al., 2005). Furthermore, circular strategies may involve asymmetric information between market players, resulting in costly screening and signalling behaviour, moral hazard, and principal agent problems (Siderius and Zink, 2023; Van Opstal et al., 2024). Finally, bargaining, monitoring, and governance costs may cause transaction and search costs between market participants, precluding the uptake of circular strategies (Hansmann, 1991; Nygaard, 2022).

### 3.2 Empirical framework

To evaluate the potential role of housing cooperatives in enabling and embedding circular strategies, we need to combine perspectives from different stakeholder groups, including housing cooperatives, providers of circular solutions, real estate actors, and experts and policymakers that have a broader perspective. It is important to capture the undocumented and implicit knowledge of stakeholders who are directly experienced with implementation barriers and possess expertise in housing cooperatives or circular housing strategies. Therefore, we conducted 23 semi-structured interviews with professionals involved in the development, operationalisation, or evaluation of housing cooperatives or housing-related circular strategies. This sample size was deemed appropriate to achieve thematic saturation (Francis et al., 2010) and to ensure representation of the most relevant stakeholder groups involved in enabling or assessing circular transitions in housing. The stakeholders were selected to reflect the full spectrum of actors relevant to circular housing transitions, including housing cooperatives (as demand-side implementers), service providers (as supply-side enablers), real estate actors (as developers and infrastructure managers), finance providers (as financial enablers), and policymakers and experts (as systemic enablers and evaluators).

Semi-structured interviews are widely recognised as an effective method for gaining an in-depth understanding of complex and multifaceted issues, capturing perceptions, opinions, and feelings or respondents underlying their behaviour (Bell et al., 2022). Semi-structured interviews are a valuable qualitative research tool, providing the flexibility to explore unexpected perspectives while maintaining a clear focus on the core research questions (Galletta, 2013). They allow us to capture positivist cognitive elements but also enables an interpretivist qualitative research approach, paying attention to subjective understandings and contextual sensitivities by interview respondents (Gehman et al., 2018).

A first groups of interview respondents was selected according to their commitment to a ‘Living Lab’ project on ‘Hybrid housing’, funded by the Flemish Government. This project brings together multiple stakeholders to explore how affordability and circularity can be aligned and integrated into alternative housing configurations. In this project, we actively screened, identified, and interviewed cooperative housing solutions in Belgium. Next, we selected service providers with expertise in delivering as-a-service solutions for solar photovoltaics, heat pumps, and kitchens. These systems are integrated into homes and as-a-services can be considered as non-default solutions in Belgium, where ownership models predominantly govern these markets. Thirdly, we selected real estate actors, finance providers, policymakers and experts that have been experimenting with housing cooperatives and circular strategies, as documented in earlier projects or referred to by interview respondents. The broad set of consortium members of the Living Lab project helped to identify stakeholders and reflect on the proposed set of interview respondents. Likewise, during the interviews we applied a snowballing sampling technique, asking interview respondents which stakeholders we should include in our sample, further validating the set of interview respondents to include in our sample. Sampling stopped when the level of data saturation was reached, meaning that further data collection and coding resulted in minimal or no new insights (Francis et al., 2010).

During the semi-structured interviews, we asked targeted follow-up questions to make implicit assumptions and tacit knowledge explicit. Therefore, we asked each interview respondent as a last question to perform a premortem analysis explicating why, by 2030, it would become clear that the combination of housing cooperatives with affordable housing and the implementation of circular strategies would turn out to be a complete failure. This technique, originating from behavioural economics (Gallop et al., 2016; Thaler and Sunstein, 2008), helps to mitigate cognitive biases among interview respondents such as confirmation bias, hindsight bias, and groupthink.

A translated version of questions and an anonymized version of the list of respondents is included in Appendix A. Interviews were conducted with informed consent via MS Teams and lasted between 50 and 85 min. A first round of interviews occurred between May and June 2024, followed by an initial analysis and feedback rounds. A second round of interviews took place between December 2024 and February 2025. All interviews were recorded, transcribed, and coded in Atlas.ti. The coding process started with a deductive coding of the interview transcripts based on circular strategies, cooperative governance, housing typologies, and stakeholder roles. Next, an inductive approach was applied to identify themes and patterns within and between data categories, and to link themes and underlying insights to the analytical framework described in Section 3.1 (Creswell and Poth, 2016). Finally, all interview respondents received a draft version of this paper to refine arguments and prevent misinterpretations and factual errors.

## 4 Results

### 4.1 Cooperative governance and circularity in housing

In Table 2, we summarize organisational advantages and disadvantages of housing cooperatives to enable and embed circularity goals, structured along cooperative governance principles.

**Table 2:** Organisational (dis)advantages of cooperative governance to implement circular strategies in housing.

ICA principle	Organisational advantages for CE	Organisational disadvantages for CE
1. Voluntary and open membership	Housing cooperatives can attract members committed to sustainability, fostering a shared vision for circular housing	Membership models may limit scalability; cooperatives rely on individuals opting in, limiting their potential for a widespread adoption of circular principles
2. Democratic member control	Enables participatory governance, allowing members to co-decide on circular strategies	Decision-making can be slower and conflict-prone, especially when balancing affordability, circular investments, and diverse member preferences
3. Member economic participation	Encourages collective investment in circular housing features Cooperatives retain value locally rather than extracting profits, aligning with long-term CE goals	Financing large-scale circular investments may be challenging (limited member investment capacity). Likewise, older cooperatives may struggle to retrofit circular solutions without external financial support.
4. Autonomy and independence	Cooperatives can prioritise long-term CE goals rather than short-term profitability, as they do not feel pressure from short-term investors	In countries where housing cooperatives are ‘niche’ players, they suffer from limited bargaining power with banks and policymakers Dependence on external regulation (e.g., VAT on renovation, cooperative legal status) can hinder circular innovation
5. Education, training, and information	Culture of knowledge sharing, enabling members to adopt circular practices Housing cooperatives can foster skill-development for members in sustainable housing maintenance	Requires ongoing investment in educational programmes, which may be deprioritised when financial and time constraints arise
6. Cooperation among cooperatives	Collaboration with other cooperatives enables scaling of best practices and joint procurement power	Lack of incentives to collaborate between cooperatives with strong mission-oriented identities, lack of network governance, and lack of standardisation among cooperatives may slow systemic change
7. Concern for community	Cooperatives are mission-driven and embedded within their communities, enabling them to generate positive externalities, including social and environmental goals in housing	Potential exclusion effects stemming from high membership engagement requirements, making it harder for less resource-full groups to participate (insider-outsider problem)

A *first* overarching advantage of cooperatives is their long-term ownership of housing stock, coupled with the alignment of ownership and tenancy, which provides clear economic incentives to invest in sustainable construction methods and energy-efficient retrofits. As put by an interview respondent: “*compared to traditional ownership – and even among other cooperatives – housing cooperatives involve an investment on a truly long term”. Secondly*, the cooperative model also encourages shared spaces and pooled resources, leading to higher use intensity of common facilities, such as kitchens, laundries, and communal tools, which decreases per capita material consumption while reducing costs. *Thirdly,* democratic decision-making within cooperatives fosters collective responsibility for maintenance and sustainability initiatives, supported by psychological mechanisms such as trust, reciprocity, and reputation, which also reduces agency costs. Likewise, given strong behavioural externalities within housing cooperatives, there is a strong incentive to educate members. *Fourthly,* by pooling purchasing power, resources, and experiences, cooperatives can lower costs and enhance financial access to durable materials and construction techniques, and service and sharing models that may be inaccessible to individual homeowners or landlords. *Finally,* cooperative governance enhances coordination among members, facilitating the establishment and enforcement of sustainability standards from the outset.

Cooperative governance also presents structural challenges that may impede the implementation of circular strategies. A *first* overarching challenge is that cooperative decision-making, while democratic, can be slow and complex, particularly when members have diverse preferences regarding affordability, circular investments, and long-term sustainability trade-offs. The dual role of cooperative members as both users and owners complicates decision-making compared to external ownership models, where governance is primarily driven by financial returns. Assortative matching among cooperative members with shared preferences and beliefs helps mitigate this challenge but can also limit the openness and inclusivity of cooperatives and even create an insider-outsider dynamic. A *second* challenge also stems from democratic governance, as it may result in a lack of in-house expertise. This could be partially resolved by cooperating between cooperatives, but also there, mission heterogeneity can impede collaboration, which can be considered as a *third* barrier. A *fourth* barrier is a lacking supporting ecosystem, resulting in higher transaction costs. This mostly applies to regions where cooperatives are relatively unknown (Bengtsson, 2024; Czischke et al., 2025; Noureldin et al., 2024). This results in underdeveloped financial services, rendering access to finance challenging as banks consider housing cooperatives as high-risk due to their non-traditional ownership structure and the collective nature of their financial obligations. Is also applies to regulatory frameworks that favour individual homeownership over alternative housing models, imposing higher VAT rates on renovations or limiting access to subsidy schemes for renewable energy.

### 4.2 The cooperative difference across housing types

In Table 3, we examine the cooperative difference when implementing circular strategies in three different housing types: single houses, apartment buildings, and cohousing projects. In *single-family housing*, cooperative models face challenges due to relatively high individual autonomy and diseconomies of scale. Moral hazard is more pronounced as there is limited peer monitoring, making it harder to ensure sustainable use of shared infrastructure or performance-based service models. Additionally, circular investments, such as modular construction or shared energy solutions, become more expensive per unit compared to multi-unit housing models, reducing their feasibility.

**Table 3:** Barriers for the uptake of circular strategies, housing types, and the cooperative difference.

	Single houses	Apartments	Cohousing
**Barriers for the uptake of…**			
Design-based strategies	High upfront cost for circular design, often unaffordable for individual homeowners Existing single-family houses are often difficult to retrofit for circularity	Building codes often favour traditional designs and traditional parking spaces	Unique cohousing projects make it harder to mainstream circular design Lack of resale market for modular cohousing units.
PSS	High customer acquisition cost (lack of economies of scale) Moral hazard among users in sharing and service models, with limited peer-control Payment risk of assets that become immovable by destination (e.g., kitchens, solar PV, …), limiting access to bank finance	Lack of functional governance of associations of co-owners Regulatory barriers on energy sharing in apartments, limiting shared PSS models	Cohousing communities may hesitate to commit to long-term PSS contracts Regulations may hinder shared ownership of (energy) infrastructure in cohousing
Sharing models	Residents of single-family houses often prefer individual access and ownership Energy and mobility sharing is often restricted and insufficiently available in rural areas	If residents do not see clear cost savings, they may not engage in sharing initiatives Existing apartments are mostly not designed for shared services (e.g., common laundry, shared storage)	Residents may resist sharing due to privacy and trust concerns Decision-making over shared assets can be prone to conflict and moral hazard
**Cooperative (dis)advantages to resolve these barriers**			
	✅ Cooperative governance can incentivise shared infrastructure and provide a market pull for circular solutions. ❌ Cooperative single-house projects suffer from diseconomies of scale in implementing circular strategies ❌ Cooperative single-house projects lack peer-monitoring on user behaviour, and cooperative members have limited social interactions to foster learning	✅ Cooperatives can organise and govern sharing models for their members and local communities ❌ In mixed-ownership apartments, cooperative models may conflict with non-member preferences	✅ Cohousing is intrinsically aligned with non-ownership and sharing models ✅ Collective decision-making fosters long-term viability of shared infrastructure.

Note: ✅ indicates where cooperatives have a comparative organisational advantage, whereas ❌ indicates a comparative disadvantage.

In *apartment buildings*, the combination of cooperative governance and economies of scale may foster the development and resource-efficient use of shared infrastructure, and the uptake of PSS and sharing models. As illustrated by a service provider: *"The collective nature of apartments is essential for us. It justifies for residents that the installation does not belong to them."* Additionally, collective contracting reduces transaction costs, simplifying the negotiation of long-term agreements for maintenance, energy services, and resource efficiency improvements. However, governance complexity increases with larger resident groups, particularly when preferences are diverse. Therefore, PSS models involving installations subject to strong and divergent user preferences, such as kitchens, are considered less suitable for cooperatives. PSS models for technical systems, such as solar PV and heat pumps, typically involve weaker and more homogeneous preferences and are therefore generally more viable in housing cooperatives. Furthermore, decision-making can be slow and prone to conflict, especially in mixed-ownership settings where cooperative members must coordinate with private landlords and non-member residents.

*Cohousing* provides the strongest alignment between cooperative governance and circularity principles, as it is inherently structured around shared ownership and collective decision-making. Residents are more likely to engage in shared services, such as mobility solutions, community energy infrastructure, and centralised resource management, due to their commitment to collaborative living. Furthermore, the behavioural externalities within cohousing communities reduce information asymmetries, supporting the viability of long-term circular service contracts. As illustrated by a respondent: *“Once people are living together, it brings people together. In these intentional communities, there are structured ways to exchange and share resources. This is common – they know and trust each other.”* However, regulatory barriers, such as restrictions on energy sharing, may complicate the implementation of circular models. Additionally, a smaller resale market for modular cohousing units may create uncertainty around future investments.

### 4.3 Housing cooperatives and stakeholder perspectives

In this section, we address different stakeholder perspectives related to circular strategies in cooperative housing and explore to which extent housing cooperatives can align stakeholder incentives, as depicted in Table 4.

**Table 4:** Stakeholder positions, main barriers to implement circular strategies, and cooperative enablers and challenges.

Stakeholders	Main barriers	Cooperative enablers	Cooperative challenges
Residents	Higher upfront costs of circular design solutions deter interest Lack of knowledge about circular benefits Preference for conventional ‘ownership’ models Myopic choice behaviour, neglecting total cost of ownership Trust issues in long-term leasing contracts Concerns over privacy and control in shared resources Potential free-rider problems in shared services	Economies of scale by bundling demand and financial resources. Education and awareness-raising via participatory design Cooperative models showcase the importance of access over ownership Long-term co-ownership model allows a lifecycle approach to investment. Community-driven leasing models provide transparency and trust Strong community governance, fostering fair use of shared spaces Improved access to social psychological mechanisms, such as reputation and reciprocity, increasing trust and cooperative behaviour	Lack of access to external funding Lack of knowledge among cooperative members Increased entry barriers, attracting converted crowds Bargaining cost of alignment, resulting in slow decision-making Monitoring and enforcement costs by members
Landlord	Split incentives: landlords bear costs, residents enjoy benefits Higher initial investments with unclear return and impact on resale values Risk of moral hazard by residents in circular models (e.g., repair, maintenance, sharing) Risk of provider bankruptcy, service failure, or contractual lock-in by PSS providers	Double role of cooperative members: residents are co-owners Cooperative investments driven by user-oriented instead of financial motivations Shared risk through cooperative contracting and governance structures Stronger bargaining power from (large-scale) cooperatives compared to individual households	Bargaining cost of alignment, resulting in slow decision-making Increased entry barriers, attracting converted crowds Monitoring and enforcement costs by members Need for economies of scale to provide countervailing power and capability development
Financial institutions	Difficulties valuing circular properties for loans (high-risk remarked value) Lack of PSS-oriented funding structures Uncertainty in financing shared assets vs. individual assets (moral hazard)	Risk pooling through cooperative contracting and governance structures Cooperative governance mechanisms may stimulate monitoring and enforcement	Higher interest rates for coops (bankruptcy laws) compared to individual citizens Switch cost to adapt credit decision processes and software to less known models, such as housing cooperatives
Suppliers Service providers	Incomplete circular supply chains and ecosystems for circular housing solutions Lack of customer experience with PSS models, leading to high customer acquisition costs Market preference for individual ownership models Moral hazard problems with service and sharing models Payment risk for assets that become immovable by destination	By bundling purchasing power, cooperatives may create a market pull for circular solutions. Given the long-term horizon of housing cooperatives, service suppliers may develop long-term partnerships Cooperatives can establish service and sharing models as ‘default’ options, nudging residents into circular strategies Cooperatives can serve as frontrunner market segments, allowing supplier to demonstrate the viability of their circular solution Cooperative governance may include sociopsychological mechanisms to monitor residential behaviour	Assortative matching of resourceful cooperative members Bargaining cost of alignment, resulting in slow decision-making
Policymakers	Regulatory frameworks (e.g., zoning laws) favour conventional building Circular housing solutions are not well-integrated into public tenders Regulatory barriers to energy sharing and leasing models Public funding schemes favour ownership models	Cooperatives may leverage their non-profit status to qualify for exemptions or special provisions Bottom-up initiatives may provide living labs to demonstrate feasibility of circular housing policies, energy sharing, and sustainable mobility solutions	Unknown model in many countries and regions Hard to make a case to prioritise cooperative over social housing, if they fail to provide affordable housing

For *residents*, cooperatives can mitigate financial, cognitive, and cultural barriers by bundling demand and financial resources, offering participatory education, and fostering trust in shared ownership and leasing models. The long-term co-ownership structure allows for lifecycle-oriented investments that prioritise sustainability over short-term savings while enabling affordable access to higher-quality materials and appliances. As illustrated by a finance provider: *“Most people overlook maintenance expenses, making it extremely difficult to convince them to pay from day one for a model that includes maintenance costs. I believe the only viable market entry is through situations where the developer is responsible for long-term management, as is the case in housing cooperatives"*. However, cooperative governance also introduces challenges, such as high entry barriers that may attract only highly motivated individuals that are willing to invest time and resources in democratic decision-making and monitoring activities. For *landlords*, the split incentive problem remains a key barrier in traditional housing models, where they bear the cost of circular investments while tenants reap the benefits. Cooperatives inherently resolve this issue, as residents are both tenants and co-owners, ensuring that incentives for sustainability investments are aligned.

*Financial institutions*, including banks and investors, often struggle to assess the value and risks of cooperative housing models, particularly when financing circular assets such as renewable energy infrastructure, modular construction, and shared services. Risk aversion towards housing cooperatives and a lack of familiarity with PSS-based models contribute to higher interest rates and limited access to capital for cooperatives compared to traditional homeowners. However, cooperatives can offer risk-pooling mechanisms and structured governance that can enhance financial stability. As claimed by a respondent: *“In Switzerland, banks reportedly approach housing cooperatives, because they acknowledge them as a highly stable investment - not only because it involves real estate, but also because the cooperative model provides confidence in proper management."*

*Service providers*, such as providers of PSS and sharing models, can benefit from cooperatives as early adopters of circular solutions, using them as first-mover markets to demonstrate the viability of new business models. Cooperatives can facilitate this by bundling demand, negotiating long-term service contracts, and embedding circularity as a default within their governance frameworks. *Policymakers* also play a crucial role in enabling cooperatives to drive circularity, particularly by reforming fiscal policies to support repair and maintenance rather than new construction. Expanding subsidy schemes for individual homeowners to include cooperative members could further enhance their capacity to implement circular strategies. However, the cooperative model remains relatively unknown in many policy contexts, making it difficult to prioritise over social housing unless it can demonstrably deliver affordability alongside sustainability. As illustrated by a respondent: *“Some civil society organisations oppose public support for housing cooperatives, advocating instead for prioritising investments in social housing."*

### 4.4 Comparative organisational (dis)advantages

Finally, we compare housing cooperatives with other housing initiators and identify their comparative organisational (dis)advantages in Table 5.

**Table 5:** Comparative organisational (dis)advantages for integrating circular principles in housing.

Housing cooperatives	Social housing associations	Public authorities	Commercial real estate developers
✅ Long-term orientation aligns with investment in durability and TCO considerations ✅ Can embed circularity principles from the start ✅ Strong community trust may enable shared resource use ❌Larger governance costs under heterogeneous membership ❌Attracting debt capital can be costly (higher financial risk perception from banks) ❌Volunteer-driven governance may slow decision-making on technical issues ❌Assortative matching of resourceful members and converted crowds	✅ Large housing portfolios may allow for economies of scale ✅ Shared spaces may reduce costs ❌Budget constraints and a strong focus on affordability may result in prioritising low-cost solutions over circularity ❌Public procurement frameworks may create barriers to adopt as-a-service models ❌Split incentives between social housing associations and tenants ❌Budget cycles may cause myopia towards TCO considerations	✅ Political power to create demonstrator projects, financial incentives, and regulations for circular solutions ❌Lack of circularity criteria in public procurement, resulting in a tendency to select lowest-cost bids ❌Political cycles reduce long-term commitment to circular projects ❌Budget cycles may cause myopia towards TCO considerations	✅ Large-scale projects can drive demand for circular solutions ✅ Some developers integrate as-a-service models to lower upfront costs, unburden maintenance concerns, and differentiate for green customers ❌High upfront costs make sustainable construction competitively unattractive ❌Value creation may exceed value capturing when adding circular solutions ❌Sales models may cause myopia towards TCO considerations

Note: ✅ indicates where cooperatives have a comparative organisational advantage, whereas ❌ indicates a comparative disadvantage.

Housing cooperatives possess structural organisational traits that help to enable and embed circular principles, as discussed throughout this paper. *Firstly*, they have a long-term perspective, just as public authorities, which aligns well with circular investment strategies. However, political and budgetary cycles often lead to short-term prioritisation in public sector decision-making. In contrast, commercial real estate developers operate under strong financial constraints that favour short-term returns, making circular investments less attractive. Social housing associations often prioritise affordability due to budgetary constraints, limiting their ability to engage in higher upfront investments for circular solutions.

*Secondly*, cooperative governance is user-driven, which may allow members to embed circularity from the outset, making service and sharing models the default option, which may render it economically viable. On the contrary, commercial developers are primarily driven by financial incentives. Public authorities and social housing associations are socially driven, focusing on affordability and sustainability, but operating within political and bureaucratic frameworks, which can reduce flexibility in adopting circular innovations.

*Thirdly*, the ability to drive systemic change through market influence varies across housing models. Housing cooperatives, despite their sustainability commitments, often operate at a relatively small scale, limiting their ability to influence suppliers and construction markets. However, in countries where this model is sufficiently established, housing cooperatives can, through collective purchasing and standard setting, generate a market pull for circular solutions. Social housing associations, due to their large portfolios, have significant potential to scale circular solutions but require structural policy support to overcome financial barriers. Public authorities can act as market enablers by integrating circular requirements into procurement and land-use policies, but their impact is often constrained by regulatory limitations. Commercial real estate developers, particularly those engaged in large-scale projects, have the capacity to standardise circular practices, but their willingness to do so depends on financial viability and regulatory pressures rather than inherent organisational priorities.

*Finally*, access to capital presents a major challenge for housing cooperatives, as banks and financial institutions often perceive them as high-risk borrowers, primarily due to their non-traditional ownership structures rather than their intrinsic organisational design. This limits their willingness to secure funding for large-scale circular renovations or new developments. Social housing associations and public authorities, on the other hand, have relatively stable funding sources but may face regulatory and budgetary constraints that prioritise cost-cutting over sustainability. Public procurement frameworks frequently favour the lowest-cost solutions, making it difficult to integrate circular principles unless specific policy incentives are introduced. Commercial real estate developers, despite having stronger access to capital, often struggle with value capture in circular investments, as they tend to operate on a sales-driven model that does not reward long-term resource efficiency.

## 5 Discussion and conclusions

### 5.1 Empirical contributions

In this study, we investigated how housing cooperatives can enable and embed circular strategies. A *first* empirical contribution stems from applying cooperative theory (Novkovic, 2008; Spear, 2000) to housing cooperatives within the context of circular economy strategies. Our findings illustrate how cooperative governance structures can enable circularity through collective decision-making, shared resource use, and long-term investment in sustainable housing. By systematically analysing the role of the ICA cooperative principles in housing cooperatives, we provide empirical evidence on how different governance mechanisms foster or hinder the implementation of circular strategies across various housing typologies.

*Secondly*, our study advances insights on aligning incentives among diverse stakeholders in circular housing. The successful implementation of circular strategies depends on collaboration among value-chain stakeholders (Asgari and Asgari, 2021), and requires a sound alignment of incentives (Van Opstal et al., 2024). In housing, this requires collaboration between a wide variety of stakeholders, including residents, financial institutions, policymakers, and service providers. Our findings indicate that cooperative governance mitigates some of the misaligned incentives present in traditional housing markets. For instance, cooperatives inherently mitigate the split-incentive problem between landlords and tenants, as residents are both co-owners and users, leading to a stronger alignment of sustainability and affordability objectives. Moreover, cooperatives can act as early adopters of circular solutions, creating a market pull for service-based business models and shared infrastructure.

*Thirdly*, from a law and economics perspective (Ben-Ner, 2018; Hansmann, 1991, 1999), this research sheds light on the comparative institutional advantages and disadvantages of cooperatives in implementing circular housing strategies. While housing cooperatives benefit from long-term investment horizons and community-driven decision-making, they also encounter financial and regulatory barriers that limit their scalability. Our analysis highlights that cooperatives may face higher transaction costs due to underdeveloped financial services and policy frameworks that favour individual homeownership.

*Fourthly*, while future research could more explicitly assess which specific circular strategies are most compatible with cooperative governance, our findings suggest several promising areas. First, design for long-term adaptability (e.g. modular construction, standardised components) aligns well with the long-term orientation and lifecycle planning inherent in cooperative housing. Second, shared infrastructure designs (e.g. centralised energy or laundry systems) benefit from cooperative governance and bundled procurement. Third, cooperatives may be well placed to implement design-for-maintenance and repair features, as their ownership structure enables collective maintenance planning and reinvestment. However, more individualised strategies, such as highly customisable interior fittings, may be less compatible due to governance complexity and diverging user preferences.

*Finally*, although this study is based on the Belgian context, the mechanisms identified, such as participatory governance, long-term ownership, collective investment structures, and stakeholder alignment, are relevant in three ways. First, they offer practical guidance for countries where cooperative housing is still emerging and where new institutional models are currently under design. Second, in regions with established cooperative sectors, these findings can support the integration of circularity objectives into existing governance frameworks. Thirdly, by focusing on its organisational design characteristics, the empirical contribution of this paper extends beyond the specific cooperative model. Public housing, co-housing schemes, and emerging hybrid models may equally benefit from applying these principles to enable and embed circular housing strategies. In this sense, the paper contributes to wider debates on how to operationalise circular housing transitions through governance innovation, even in contexts where formal cooperative structures are lacking or unsupported.

### 5.2 Policy implications

Policymakers and cooperative federations should recognise that many housing cooperatives do not inherently prioritise circularity. Affordability and basic functionality often dominate cooperative agendas, particularly in contexts with constrained resources or limited governance capacity. Therefore, circular interventions must be made not only technically feasible and financially viable but also desirable from the perspective of cooperative members (Bocken et al., 2022). Building on this, the following sections outline how housing cooperatives can capitalise on their organisational comparative advantages and how policy support can address systemic barriers and create enabling conditions at multiple governance levels.

#### 5.2.1 Policy implications for housing cooperatives

Housing cooperatives have the potential to act as frontrunners in embedding circularity into housing models, but they must align their governance structures, financial models, and stakeholder engagement practices to maximise their impact. One key policy implication is the opportunity to integrate CE principles into cooperative governance and business models from the outset. By clearly defining CE objectives in their bylaws, cooperatives can establish internal incentives that encourage lifecycle thinking and prevent short-term cost-cutting measures that undermine sustainability. It also sets CE as the default option, reducing future bargaining costs among members and attracting new members who prioritise sustainability. This transforms the adoption of circular solutions from a prisoners’ dilemma into a coordination game, making switching to the non-circular option costly in both monetary and social terms (Bowles and Gintis, 1998). Nevertheless, housing cooperatives face challenges in motivating their members to engage in costly monitoring and enforcement efforts, as members may have an incentive to freeride (Hansmann, 1991).

While aligned preferences reduce internal governance costs, assortative matching mechanisms may turn housing cooperatives into exclusive enclaves (Ferreri and Vidal, 2022). Therefore, governance should be structured to balance inclusivity with efficient decision-making, safeguarding affordability when considering circular solutions. However, cooperatives must also avoid goal overload (Greve, 2008) stemming from a multitude of missions, resulting in excessive complexity that may hinder implementation of any goal. Importantly, circularity is a pathway to sustainability, not a destination (Chen, 2021). Nevertheless, providing education and training on repair, maintenance, and shared resource governance can enhance both member participation and long-term commitment to CE principles.

Next, housing cooperatives should collaborate to benefit from economies of scale. Internal supply-side economies of scale arise when housing cooperatives coordinate procurement strategies and standardise circular construction processes across multiple projects. Cooperatives should safeguard that cost savings involved do not lead to rebound effects, as established by Shinde et al. (2022) who found that cooperative residents tend to spend the ‘extra’ income on housing and travel expenditures, jeopardizing environmental goals. External supply-side economies of scale emerge when housing cooperatives collaborate to develop supportive ecosystems, such as tailored financial products and enabling policy frameworks. To achieve this, cooperation among cooperatives is essential, requiring a focus on shared objectives rather than differences. This could result in ‘secondary’ cooperatives, servicing tenant-managed ‘primary’ cooperatives with professional expertise (Lang et al., 2020), or in partnerships between large cooperatives and new cooperative initiatives (Lang and Stoeger, 2018). However, strong mission-driven identities within individual cooperatives can hinder collaboration. This mechanism resembles the “*Life of Brian”* problem (Baturo and Mikhaylov, 2013), reflecting how groups prioritise their own belief systems over broader collective goals, leading to a lack of incentive compatibility for cooperation. Finally, housing cooperatives can foster dynamic economies of scale by sharing knowledge and developing best practices through collaboration within and across borders.

#### 5.2.2 Policy implications for governments

Housing cooperatives offer a bottom-up approach to circularity, but their success largely depends on the existence of supportive policies and ecosystems (Aernouts and Ryckewaert, 2019; Ahedo et al., 2023). As Elster (1989) famously questioned – and as Bengtsson (2024) applies to housing cooperatives – *if cooperatives are so desirable, why are there so few of them?* This paradox highlights the structural and policy barriers that limit their widespread adoption. As stressed by Ferreri & Vidal (2022), the role of the state across different policy levels is a crucial determinant in whether housing cooperatives remain a ‘niche’ solution or scale into a broader movement. Consequently, policymakers at different government levels play a role in enabling cooperatives to contribute to affordable and circular housing.

*Municipal governments* can support the uptake of circular strategies by housing cooperatives by providing access to land, streamlining permitting processes, and incentivising circular building practices. Offering long-term land leases or reduced land costs for cooperatives that integrate CE principles can improve feasibility, as it unburdens housing cooperatives from one of their largest barriers, improving mental bandwidth to consider affordability and circularity in their project. Zoning regulations and permitting processes should be adjusted to recognise alternative housing models that prioritise shared ownership, energy efficiency, and material reuse. Furthermore, cities can serve as living labs for cooperative circular housing initiatives, facilitating pilot projects that demonstrate feasibility and scalability (Galle et al., 2021), supporting capacity building and partnership development, as well as providing government guarantees to back loans of housing cooperatives.

At the *regional and national levels*, fiscal policies should be revised to create an equal playing field between cooperative and individual ownership models. Firstly, this includes ensuring that renovation and deconstruction activities undertaken by cooperatives benefit from the same (reduced) VAT rates and expanding subsidy schemes from individual owner-based models to access-based business models in cooperatives. Secondly, regulatory reforms could also consider cooperative structures for investments in circularity and energy efficiency to resolve the split incentive problem between landlords and tenants. Thirdly*,* a formal legal recognition of housing cooperatives as a distinct category - separate from traditional rental and ownership models - can pave the way for tailored financial and governance frameworks. Finally*,* knowledge transfer and capacity building among cooperatives should also be actively supported, facilitating collaboration between established cooperatives and new initiatives to share best practices and strengthen the cooperative ecosystem.

At the *European and international levels*, knowledge-sharing platforms that document best practices and provide technical guidance for integrating CE principles into cooperative housing should be supported. Establishing EU-wide regulatory guidelines for cooperative housing models can enhance policy coherence and improve legal recognition across member states. Funding mechanisms under the European Green Deal, Horizon Europe, and regional development programmes could explicitly support cooperative-led circular housing initiatives. Financial instruments from the European Investment Bank and regional development banks could foster shared ownership structures and circular financing models, making it easier for cooperatives to access affordable capital for long-term investments. Finally, cooperative housing should be acknowledged as a key instrument in delivering on the Sustainable Development Goals (SDGs), particularly SDG 11 on sustainable cities and communities.

### 5.3 Limitations and recommendations for further research

This study provides an initial exploration of how housing cooperatives can enable and embed circular strategies, highlighting both their comparative organisational advantages and structural challenges. However, some limitations must be acknowledged.

*First*, our findings are mostly drawn from the context of Belgium, which has a frontrunner position implementing a circular economy (Claudio-Quiroga and Poza, 2024; D’Adamo et al., 2024) but lacks a strong tradition in housing cooperatives. While countries with well-established cooperative housing sectors have been extensively studied and documented, those where cooperatives remain a small niche are substantially understudied. This leaves it unclear to what extent observed advantages and challenges stem from the intrinsic design qualities of cooperatives or from broader institutional path dependencies. Examining cooperatives in a context where they remain a niche model allows for a focus on market-, and technology-agnostic organisational design aspects, reducing potential bias from specific national policies or historical contingencies. Nevertheless, future research should explore both regions with longer traditions as regions lacking experience with housing cooperatives to examine whether the findings hold in different institutional contexts.

A *second* limitation, stemming from the first, is that this study primarily draws on perspectives from professionals involved in the development, operationalisation, or evaluation of housing cooperatives and circular housing strategies. While this approach enables an analysis of organisational and policy-level factors, it does not include direct input from current residents. Given the early stage of many cooperative initiatives in Belgium, respondents often relied on sociotechnical imaginaries about prospective users, owners, and ecosystem players, similar to research on other complex sociotechnical systems such as smart grids (Kojonsaari and Palm, 2023; Van Opstal et al., 2025b). Future research should incorporate perspectives from cooperative residents to examine how governance structures influence circular decision-making in practice.

*Finally*, further empirical studies are needed to measure the actual differences between housing cooperatives and traditional housing models in enabling circular strategies. Quantitative research could help assess the impact of cooperative governance on circular investments and the adoption of PSS and sharing models. However, such studies should carefully account for potential selection biases. Housing cooperatives may attract a resource-rich subset of the population, particularly individuals with a strong commitment to sustainability or higher levels of social and financial capital. This creates endogeneity risks in statistical inference, making it difficult to determine whether observed differences result from cooperative governance structures or the characteristics of the members themselves, generating biased findings on the potential of housing cooperatives to a circular economy and society.

## CRediT authorship contribution statement

**Wim Van Opstal:** Writing – review & editing, Writing – original draft, Validation, Resources, Methodology, Investigation, Formal analysis, Data curation, Conceptualization. **Nancy Bocken:** Writing – review & editing, Validation. **Jan Brusselaers:** Writing – review & editing, Validation.

## Declaration of competing interest

The authors declare that they have no known competing financial interests or personal relationships that could have appeared to influence the work reported in this paper.

## Figures and Tables

**Table A.1:** Interview respondents.

ID	Position	Stakeholder type	Date
1	Founder	Housing cooperative	May 14, 2024
2	Staff member	Finance provider	May 14, 2024
3	Staff member	Intermunicipal organisation	May 15, 2024
4	Staff member	Municipality	May 16, 2024
5	Staff member	Housing cooperative	May 17, 2024
6	Founder	Housing cooperative	May 21, 2024
7	Founder	Housing cooperative	May 21, 2024
8	Founder / CEO	PSS provider	May 22, 2024
9	CEO	Finance provider	May 24, 2024
10	Staff member	Commercial real estate	June 5, 2024
11	Staff member	Commercial real estate	June 6, 2024
12	Founder / CEO	PSS provider	December 9, 2024
13	Expert	University	December 13, 2024
14	CEO	Housing cooperative	December 13, 2024
15	Founder	PSS provider	December 17, 2024
16	Founder	PSS provider	January 7, 2025
17	Founder / CEO	PSS provider	January 8, 2025
18	Founder / CEO	PSS provider	January 8, 2025
19	Staff member	Intermunicipal organisation	January 14, 2025
20	Independent expert	Consultancy	January 16, 2025
21	Founder	Housing cooperative	January 20, 2025
22	Staff member	Consultancy	January 28, 2025
23	Staff member	Municipality	February 11, 2025

## Data Availability

Data will be made available on request.

## Appendix A

In this appendix, we present an anonymised overview of interview respondents (Table A.1) and a translated (from Dutch) version of the interview questionnaires.

Interview questionnaire respondents 1 – 11 (translated from Dutch):

1. What are your experiences with (combinations) of legal persons to organise cooperative housing, while aiming for affordable and sustainable housing?
2. What are, according to you, the critical traits of legal persons (including cooperatives) to ensure affordable and environmentally sustainable housing?
3. What is needed to support housing cooperatives to enable affordability and environmentally sustainability?
4. If there would be a legal status for housing cooperatives, what would be critical design and implementation conditions?
5. What are cases of housing cooperatives we should look at, and why?
6. How can we enable and embed circularity goals in cooperative housing solutions?
7. What are crucial legal, organisational, and financial design considerations for PSS models in housing, from the perspective of users/residents, the cooperative, service providers
8. Regarding funding, what are crucial legal, organisational, and financial design considerations from users/residents, the cooperative, capital providers
9. By 2030, it is clear that the combination of housing cooperatives with affordable housing and the implementation of circular strategies turns out to be a complete failure. How did that happen? (premortem question).

Interview questionnaire respondents 12 – 23 (translated from Dutch):

1. What are your experiences with housing cooperatives and circular strategies?
  a. What was your role in this experience?
  b. What were reasons (not) to implement circular strategies?
2. What are key governance parameters for housing cooperatives to be economically, environmentally, and socially sustainable?
3. What are key organisational drivers to enable/embed circular strategies in housing?
4. What are key barriers and drivers to combine affordability and environmental sustainability?
  a. Depending on housing type (individual, collective, cohousing)?
  b. Depending on the organisational identity of the initiator (housing cooperatives, real estate developers, social housing associations, etc.)?
  c. Depending on the stakeholder position (residents, landlords, external investors, social housing associations, public administrations)?
5. What are key barriers and drivers for sustainable housing cooperatives?
  a. Scaling and replication
  b. Funding
  c. Legal/policy
  d. Other boundary conditions
6. By 2030, it is clear that the combination of housing cooperatives with affordable housing and the implementation of circular strategies turns out to be a complete failure. How did that happen? (premortem question).
